# Peptide Dendrimer-Based Antibacterial Agents: Synthesis and Applications

**DOI:** 10.1021/acsinfecdis.3c00624

**Published:** 2024-03-01

**Authors:** Suchita Paul, Sandeep Verma, Yu-Chie Chen

**Affiliations:** † Institute of Semiconductor Technology, National Yang Ming Chiao Tung University, Hsinchu 300, Taiwan; ‡ Department of Chemistry, Indian Institute of Technology Kanpur, Kanpur 208016, Uttar Pradesh, India; § Gangwal School of Medical Sciences and Technology, Indian Institute of Technology Kanpur, Kanpur 208016, Uttar Pradesh, India; ∥ Department of Applied Chemistry, National Yang Ming Chiao Tung University, Hsinchu 300, Taiwan

**Keywords:** antibacterial agents, antibacterial resistance, click chemistry, dendrimers, peptide dendrimers

## Abstract

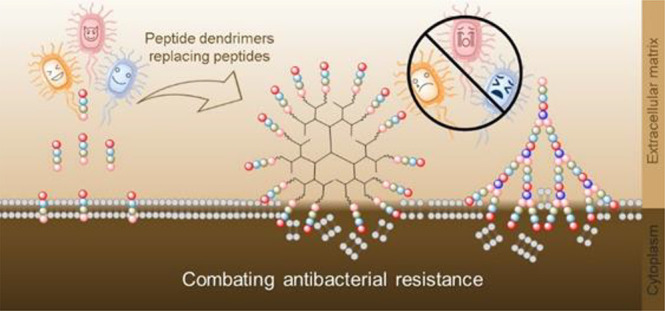

Pathogenic bacteria cause the deaths of millions of people every year. With the development of antibiotics, hundreds and thousands of people’s lives have been saved. Nevertheless, bacteria can develop resistance to antibiotics, rendering them insensitive to antibiotics over time. Peptides containing specific amino acids can be used as antibacterial agents; however, they can be easily degraded by proteases *in vivo*. To address these issues, branched peptide dendrimers are now being considered as good antibacterial agents due to their high efficacy, resistance to protease degradation, and low cytotoxicity. The ease with which peptide dendrimers can be synthesized and modified makes them accessible for use in various biological and nonbiological fields. That is, peptide dendrimers hold a promising future as antibacterial agents with prolonged efficacy without bacterial resistance development. Their *in vivo* stability and multivalence allow them to effectively target multi-drug-resistant strains and prevent biofilm formation. Thus, it is interesting to have an overview of the development and applications of peptide dendrimers in antibacterial research, including the possibility of employing machine learning approaches for the design of AMPs and dendrimers. This review summarizes the synthesis and applications of peptide dendrimers as antibacterial agents. The challenges and perspectives of using peptide dendrimers as the antibacterial agents are also discussed.

## Development of Resistance in Bacteria

**Figure 1:**
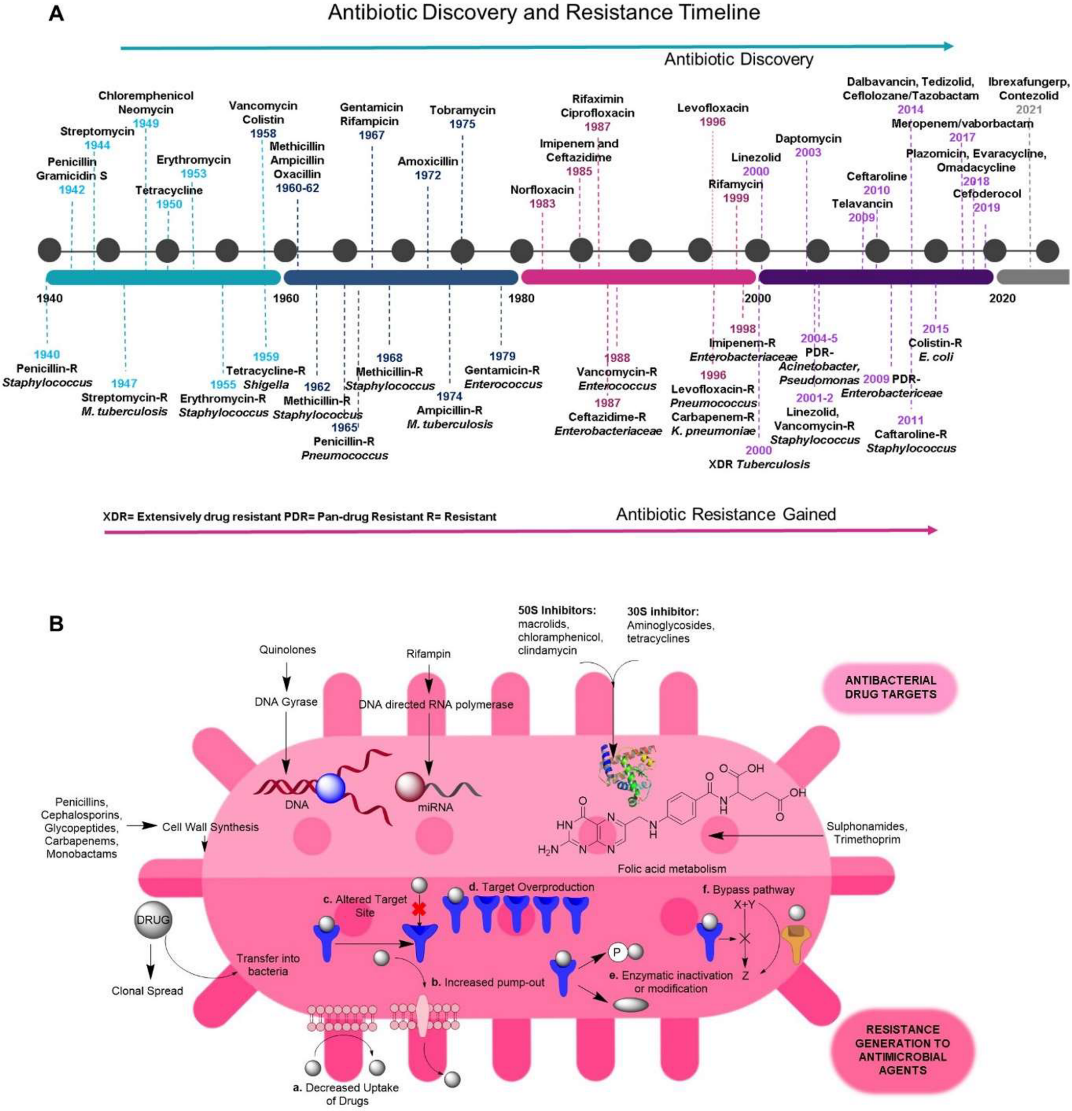
(A) Timeline of the development of antibiotics and the emergence of drug-resistant bacteria over the years.^7,11^ (B) Typical antibacterial drug targets and the pathways for the generation of drug resistance.^8,12−17^ (modifications have been made from the literature^8,11^ to bring the content up-to-date).

Bacterial infections have long posed a threat to human health and continue to be a major source of death worldwide despite decades of research. This is due to the inherent ability of bacteria to become resistant to new classes of antibiotics. There are pathogenic multi-drug-resistant (MDR) bacteria that have acquired resistance to multiple antimicrobial categories, extensively drug-resistant (XDR) bacteria that are susceptible to only one or two antimicrobial drug categories, and pan-drug-resistant (PDR) bacteria that are resistant to all clinically available drugs.^1−3^ According to a World Health Organization report in 2022, about 1.27 million deaths are caused due to bacterial antimicrobial resistance around the world.^4^ Even though the development of drug resistance in pathogenic bacteria is a natural process, various man-made variables, such as the misuse and overuse of antimicrobials, a lack of cleanliness and sanitation, the absence of sufficient health care facilities, clean water, and waste management, to name a few, play significant roles in its development.^5,6^ Despite the fact that several antimicrobial drugs are approved for clinical use each year^7^ (Figure 1A), as these new drugs are simple modifications of existing drugs, they do not appear to affect XDR or PDR bacterial strains. It was because the molecular targets or mechanisms of action^8^ (Figure 1B) of these newly developed antibiotics are identical to those of existing drugs, for which resistance mechanisms have been well established.^9,10^ The complexity of the mechanism of action, potency, and efficacy factors (duration of action or concentration), will determine whether a novel antibiotic can gain resistance or not.^8^

## General Antibacterial Drugs

Common antibiotics or small molecule medications usually have molecular weights of ∼500 Da.^18^ They are metabolically stable, orally accessible, and inexpensive.^19,20^ Antibodies, on the other hand, are protein-based therapeutic molecules that are substantially larger in size (∼150 000 Da), highly selective, and effective, but they are physiologically unstable, less permeable, and often elicit an immune response. As a result, peptide-based antimicrobials with a chain length of approximately 50 amino acids (∼6000 Da) are an alternative option, falling between small molecule drugs and large proteins in terms of size. For instance, glycopeptide-based antibiotics are small enough to reach to intracellular targets without sacrificing their affinity or specificity.^21,22^ Their main mode of action is the prevention of cell wall formation by hindering the synthesis of the peptidoglycan layer, inhibiting the subsequent transpeptidation stage and ultimately leading to bacterial cell death.^23,24^ The United States Food and Drug Administration (FDA) has approved several glycopeptide-based antibiotics for clinical therapy over the years. Some notable examples include vancomycin,^25^ gramicidin,^26^ polymyxin B, colistin,^27^ oritavancin,^28^ dalbavancin,^29^ telavancin,^30^ and daptomycin.^31^ Most of these FDA approved drugs, along with other known antimicrobial peptides (AMPs), are actually obtained from nature.^32^ According to the antimicrobial peptide database (APD3),^33^ 60% of the known AMPs fall under the category of antibacterial peptides. Among previously mentioned FDA approved antibacterial drugs, vancomycin was isolated from *Streptomyces orientalis*(34) and Gramicidin A is produced by *Bacillus brevis*,^35^ whereas polymyxin B and colistin were isolated from *Bacillus polymyxa*.^36^ Both prokaryotic and eukaryotic organisms are known to produce their own set of AMPs which protect them from external sources of infections, and they are called host defense peptides (HDPs).^37^

## Host Defense Peptides

Animals such as mammals, amphibians, and invertebrates are capable of producing HDPs on their own. Mammals such as humans, rats, sheep, or cattle can produce cathelicidins and defensins, which protect them from foreign invasions.^38−42^ Cathelicidins disrupt the cell membrane by engaging in a carpet-like antibacterial mechanism, forming micelles and leaking intracellular contents.^43^ Defensins are cysteine-rich peptides which interactively change the structural morphology in Gram-negative bacteria.^44^ Frogs can produce HDPs including magainins,^45,46^ caerulein precursor fragments (CPFs),^47,48^ xenopsin precursor fragments (XPFs),^49^ hymenochirins, and pseudohymenochirins,^50^ which possess antibacterial properties. They are essentially pore forming peptides which target the lipid matrix, causing membrane disruption and leakage of intracellular contents.^51−53^ Invertebrates also can produce several forms of defensins,^54,55^ cecropins,^56^ and crustins,^57,58^ whereas insects like drosophila flies,^59^ silkworms,^60,61^ and bees^62^ can generate HDPs, ensuring their excellent adaptability. Their antibacterial activities range from membrane permeabilization and leakage to interfering with gene transcription or preventing biofilm formation.^63−65^ Plants produce cysteine-rich thionins,^66^ defensins,^67−69^ snakins,^70,71^ α-hairpinins,^72,73^ and knottins.^74,75^ Thionins disrupt membranes,^76,77^ while cysteine-rich snakins deregulate microbial gene expression in order to prevent infection in plants.^71^ Knottins have extensive interactive and lytic ability toward membrane channels such as acid-sensing channels, K^+^ channels, or Na^+^ channels via both hydrophobic and ionic interactions.^78^ Bacteria are known to produce HDPs such as lantibiotics,^79,80^ bacteriocins,^81,82^ colicins,^83,84^ and microcins,^85−87^ which can fight against other bacteria of similar species. Bacteriocins disrupt DNA structure as well as the permeability and integrity of the cell membrane, causing leakage of intracellular biomolecules to render cells abnormally distorted.^88^ Colicins and microcins can degrade the peptidoglycan layer and phosphatase activity as well as RNAase and DNAase activity.^89^

## General Characteristics of AMPs

Most AMPs have certain similar features, which are the inclusion of both ionic and hydrophobic amino acids, giving a characteristic amphiphilic nature to AMPs (Table 1).^102^ Given that bacterial membrane has an overall negative charge, the inclusion of cationic amino acids such as arginine and lysine greatly enhances the antibacterial property of these peptides, as it helps these peptides to electrostatically bind to the bacterial membrane.^103^ Histidine can also be used in place of lysine and arginine, even though it is less effective.^104^ Tryptophan and proline on the other hand have good affinity toward the lipid bilayer, which plays a critical role in killing the bacteria. Tryptophan can cause protein misfolding, whereas proline can interact with the 70S ribosome and disrupt protein synthesis.^105,106^ Predominance of leucine and other hydrophobic amino acids is often observed in HDPs. These hydrophobic amino acids give HDPs their amphiphilic nature^107^ and help in the improvement of the insertion of HDPs into the bacterial lipid membrane for interactive membrane disruption.^108,109^ Both these cationic and aromatic amino acids can have hydrogen bonding interactions with the bacterial membrane, leading to destabilizing the lipid bilayer and helping in membrane lysis.^110^ Moreover, many HDPs contain cysteines, resulting in the formation of disulfide bonds and giving the AMPs a chemically, proteolytically, and thermally stable folded structure.^71^ Specially, crustin from crustaceans and plant HDPs are rich in cysteines (Table 1).^111,112^ These amino acids and features mentioned above are often used while designing AMPs.

**Table 1:**
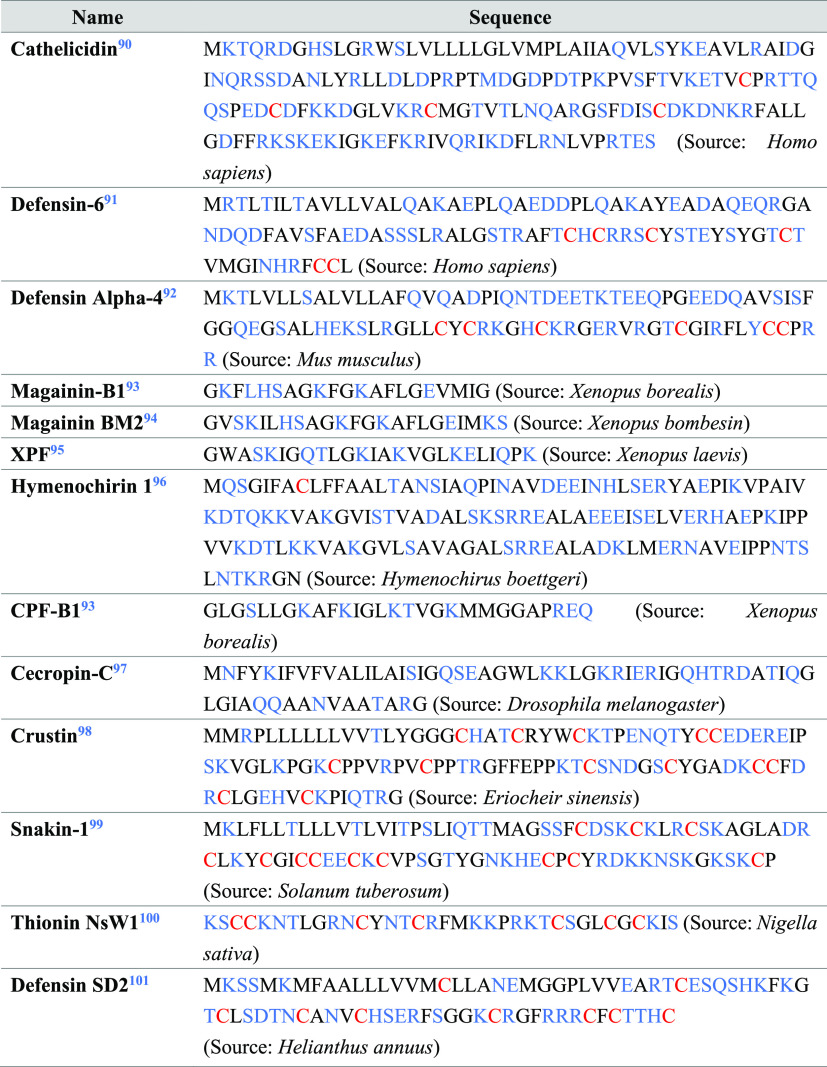
Examples of Naturally Occurring HDPs and Their Amino Acid Sequences^a^

a Color code: Hydrophilic amino acids, hydrophobic amino acids, and cysteine are marked in blue, black, and red, respectively.^90−101^

## Synthetic AMPs

Natural AMPs, however, often have problems such as high toxicity, low stability, and low salt tolerance when they are used in other organisms. Most of the time, their activity is affected by physicochemical parameters and may become inactive under certain circumstances.^113^ Hence, synthetic AMPs (SAMPs) came into the picture. SAMPs, having good to excellent antibacterial activity, are often prepared by modifying AMPs.^114^ A vast database classifies SAMPs into two types: (1) peptides that are synthesized by modifying existing peptides/proteins and (2) peptides that are completely designed from scratch. SAMPs obtained through modifications of existing proteins/peptides have a clear set of advantages such as the gain of functions (by incorporation of the required peptide sequence for a particular type of function) and reduced toxicity, as well as allergenicity.^115^ For example, the antibacterial peptide MoCBP_3_–PepIII was synthesized by modifying the protein MoCBP_3_, obtained from *Moringa oleifera* seeds, which inherently lacks any antibacterial activity.^116^ Knowing the structure–activity relationship (SAR) greatly assists the design of peptides with a broad spectrum of activity.^117^ Modifications in AMPs that have shown improved activities include substitution of amino acids, truncation, hybridization, and cyclization.^118,119^ Hybridization is a way of combining fragments from different naturally occurring AMPs to adjust the SARs of the peptide, thereby generating properties that are beneficial. For example, combining an AMP possessing high activity and high toxicity with an AMP possessing low toxicity and low activity can lead to the generation of a new chimeric AMP with high activity and low toxicity. This approach proves useful in the long run if we know exactly about the specific fragment responsible for each individual property.^119^ For instance, KR-12, the smallest antibacterial peptide from human LL-37, was converted to KL-12 by replacing all hydrophobic and hydrophilic groups with leucine and lysine to improve its antibacterial activity. GLK-19, which was designed using just glycine, leucine, and lysine, has better antibacterial activity against *E. coli* than LL-37.^120^

## Advantages and Disadvantages of Antibacterial Peptides

However, using peptides as antibacterial drugs has its advantages and disadvantages. Peptides have high efficacy and specificity, meaning that even a small dose of administration of the drug can show reliable antibacterial activity. Furthermore, they remain active against microorganisms that show resistance to conventional antibiotics.^121^ This also suggests that the required dose of antibacterial peptides is relatively low compared to other organic drugs. Antibacterial peptides have low cytotoxicity *in vivo*, primarily due to their highly specific mode of action, which does not target mammalian cells. Low *in vivo* accumulation due to short half-lives also reduces cytotoxicity. Given that peptides adopt similar tertiary structure to proteins within the human body, they do not trigger any immune response.^122,123^ However, their short lifespan can also lead to low bioavailability, even though they have high stability *in vitro* due to proteolysis by endogenous proteases that break down peptide bonds. To prevent this problem, new formulations need to be created to ensure proper drug administration, such as transdermal injection, inhalation, and the use of biodegradable polymers. Apart from these biological problems, the cost of synthesis of these peptides can be quite high, not to mention the challenges associated with ready availability of reagents and equipment for purification, which can cause major problems.^124−126^

## Machine Learning Approaches toward Designing AMPs

To tackle such shortcomings and reduce the cost of production by eliminating the synthesis of hemolytic, noneffective, and unstable peptides, machine learning models have been investigated in recent days for designing AMPs that can be both time-saving and cost-effective.^127^ Machine learning uses a plethora of peptide databases^128^ that have been categorized based on various criteria to be used as training sets for machine learning programs.^129^ These databases help researchers optimize known AMP sequences and their physicochemical parameters since it is known that α-helical peptides are known for their membrane permeation abilities while the charge distribution in the peptide helps in pore formation.^130,131^ Some of the available databases are the Antimicrobial Peptide Database,^33,132^ Collection of Antimicrobial Peptides,^133,134^ and Linking Antimicrobial Peptides,^135,136^ which fall under the category of general peptide databases.^137^ Other examples of specific databases^137^ include Hemolytik (containing hemolytic peptides),^138^ Defensins Knowledgebase (containing defensins),^139^ yet another Database of Antimicrobial Peptides (containing antibacterial peptides),^140^ Biofilm-Active AMPs (containing antibiofilm peptides),^141^ etc. Models such as k-nearest neighbor,^142−144^ support vector machine,^145−147^ recurrent neural network^148,149^ and artificial neural network^150−152^ are often used in the machine learning strategies. Machine learning has been used to either predict the AMP sequence or design a peptide with a particular activity or target.^153^ Moreover, it has gained popularity to predict and design AMPs with minimal therapeutic complications.^154^ The use of previous knowledge from ample examples available online will contribute to the growth of this field of machine-learning-based peptide design. The machine-learning-based strategies can assist researchers in identifying peptide targets by looking at the sequence of the peptide or vice versa.

## AMP-Based Nanomaterials

Another possibility of modification that has been explored in recent years is conjugating peptides on metal-based nanoparticles mostly made up of gold, silver, or iron oxide, with or without magnetic properties.^155−157^ One nanoparticle can be capped to several peptide molecules, giving a multivalent nature to the conjugates, which increases their antibacterial efficacy by several folds.^158,159^ If the nanoparticles are magnetic in nature, it makes synthesis as well as purification much easier compared to conventional methods of peptide synthesis.^160,161^ Magnetic separation of bacteria using peptide conjugated magnetic nanoparticles based on affinity binding is a well explored field of research.^162,163^ However, the toxic nature of metal nanoparticles limits its usage in *in vivo* applications of these metal–peptide conjugates.^164−166^ Hence organic-based cores are preferred, like conjugating AMPs onto fullerenes, which are carbon-based macromolecules.^167,168^ AMPs are also conjugated onto graphene oxide nanoparticles to get multivalent peptide conjugates with improved antibacterial efficacy.^169,170^ Carbon- or organic-polymer-based nanotubes are also being explored for the purpose of conjugating multiple peptides onto their surface to get multivalent, biocompatible antibacterial agents for *in vivo* applications.^171,172^ Such modifications need to be further explored.

## Brief History of Dendrimers

Dendrimers are macromolecules that have gained their recognition as versatile, well-defined, monodisperse, and derivatizable compounds, with impressive applications in both biological and nonbiological fields.^173^ They are highly ordered and hyperbranched polymeric molecules with controlled molecular weights. Their terminal polyvalency helps in the synergistic enhancement of their particular applications.^174^ The name “dendrimer” is a combination of two Greek words, “dendron” meaning tree and “meros” meaning parts. Each dendrimer mainly consists of a core, an inner shell, and an outer shell. Th addition of each layer of inner and outer shells leads to an increase in dendrimer generation (G0, G1, G3, and so on).^175,176^ The first dendrimer ever synthesized was by Vogtle in 1978.^177^ However, peptide dendrimers gained popularity when Tomalia synthesized starburst dendrimer-like macromolecules called poly(amidoamine) (PAMAM) through the alternate repetitive addition of methyl acrylate and ethylene diamine in 1985.^178^ A new class of biologically available peptide-based dendrimers, i.e., poly-l-lysine (PLL), which became the first ever known synthesized peptide dendrimer, was first synthesized by Dendewalter and co-workers in 1981.^178−181^

## Peptide Dendrimers

Peptide dendrimers are essentially large and branched polymeric structures, which are mostly made up of amino acids or short peptide chains having dendron-like moieties. Peptide dendrimers have the innate ability to mimic proteins and enzymes, leading to very low toxicity *in vivo*.^182,183^ Also, the absence of long linear and branched peptide chains with increased steric hindrance helps to escape proteolysis, providing them with improved stability and bioavailability compared to normal antibacterial peptides *in vivo*.^184^ Due to their dense and multivalent structures, even lower concentrations of peptide dendrimers are able to show excellent antibacterial properties.^185,186^ This multimeric nature gives peptide dendrimers improved sensitivity and enhanced surface binding abilities compared to their linear short peptide counterparts.^187−189^ Thus, peptide dendrimers are quickly gaining importance as viable antibacterial agents, as researchers are hopeful that they might be able to address the ever-growing problem of antibacterial resistance worldwide.^183^

## Versatility of Peptide Dendrimers

Synthesizing branching peptides or grafting peptides onto the surface of traditional dendrimers, such as PAMAM or PLL, would result in the production of globular protein-like, polyvalent, highly biocompatible, and low cytotoxic structures, which have increased resistance to proteolysis.^190^ In comparison to traditional dendrimers, peptide dendrimers have improved biodegradability.^184^ Their highly branched structures, large molecular weights, and abundance in surface functional groups result in multivalency and superior biocompatibility^191^ compared to traditional dendrimers. Linear and cyclic peptides generally fail to show prolonged stability and efficacy.^192−195^ However, branched peptides are less prone to proteolytic degradation compared to linear peptides due to the steric hindrance generated by the branches, making it difficult for serum proteases to act upon them.^184^ Peptide dendrimers having multiple functional sites such as multivalent interactions are exploited to develop novel medications and research reagents.^187^ The versatile structures and functions of peptide dendrimers poses a vast range of opportunities for applications in various biological fields^196−198^ and nonbiological fields.^199−201^ Apart from antibacterial studies, biological applications also include their use as anticancer agents,^198,202,203^ antiviral agents,^204^ drug delivery, controlled drug release,^196,205^ gene delivery,^197,206^ transfection,^207^ and protein mimics.^208,209^ Nonbiological uses of glycopeptide dendrimers include catalysis,^210,211^ hydrolysis,^200^ and aldolase-like activity.^201^ This proves the vast area of potential applications for these peptide dendrimers.

## Classification of Peptide Dendrimers

**Figure 2:**
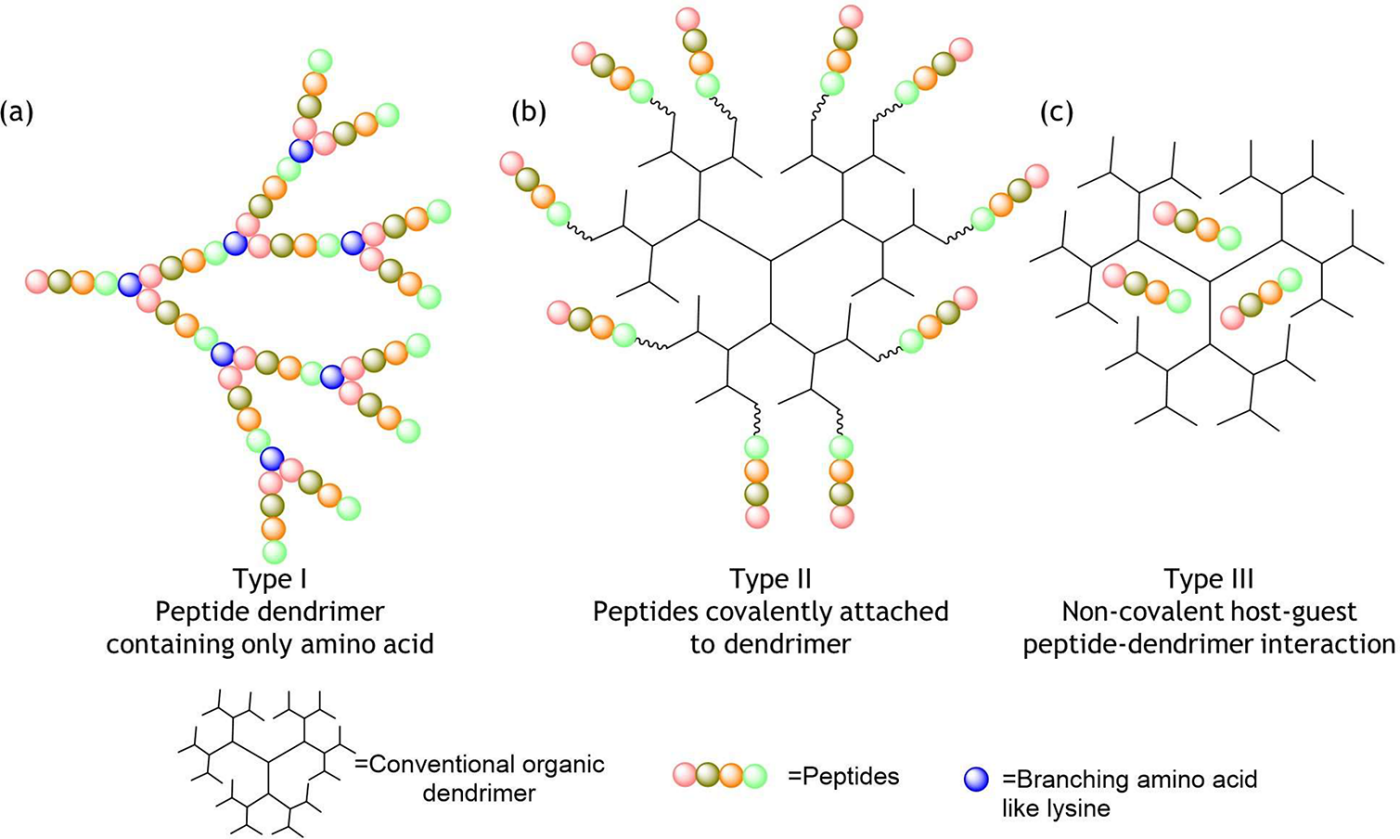
Cartoon illustrations of the chemical structures of (a) type I, (b) II, and (c) III peptide dendrimers.

Peptide dendrimers can be classified into types I, II, and III based on the nature of their chemical bonding.^212^ In type I, the branching framework of the dendrimer consists of amino acids or peptides (Figure 2a). In type II, peptides are covalently attached to the surface of an organic dendrimer core (Figure 2b). Both type I and type II of peptide dendrimers fall under the category of covalent-type bonding, since the peptides are covalently attached to dendrimers or to other peptides. In type III, however, peptides are noncovalently encapsulated within a classical organic dendrimer in a host–guest fashion, where the dendrimer acts as a host and the peptide acts as a guest (Figure 2c).

## Synthesis of Peptide Dendrimers

Like conventional dendrimers, peptide dendrimers can be synthesized via both convergent and divergent methods of synthesis. First is solid-phase peptide synthesis (SPPS). SPPS was first discovered by the Nobel prize winner Merrifield in the 1980s.^213^ In SPPS, an excess of reagents was used to synthesize peptides by attaching the C-terminal to a polymeric resin until completion.^214−216^ This SPPS approach can also be used to synthesize peptide dendrimers via divergent and convergent methods, where different protected peptide fragments are joined together to form the complete peptide dendrimer.^217,218^ Second is the general solution-phase synthesis of peptides or organic compounds. One advantage that solution-phase synthesis has over solid-phase synthesis is that intermediates can be isolated immediately after each reaction step, which reduces the possibility of unwanted reaction products. However, the purification and characterization processes can be time-consuming, and the synthesis itself may become difficult if the solubility issue arises.^219,220^ Third is the combination of both solid-phase and solution-phase synthesis, where protected peptide fragments are first created by the solid-phase approach, purified and characterized, and finally combined via the solution-phase strategy. This process gives great control over the number of side products formed. However, strong coupling agents for this strategy still need to be developed.^221−224^

## Multiple Antigenic Peptide and Various Ligation Methods

**Figure 3:**
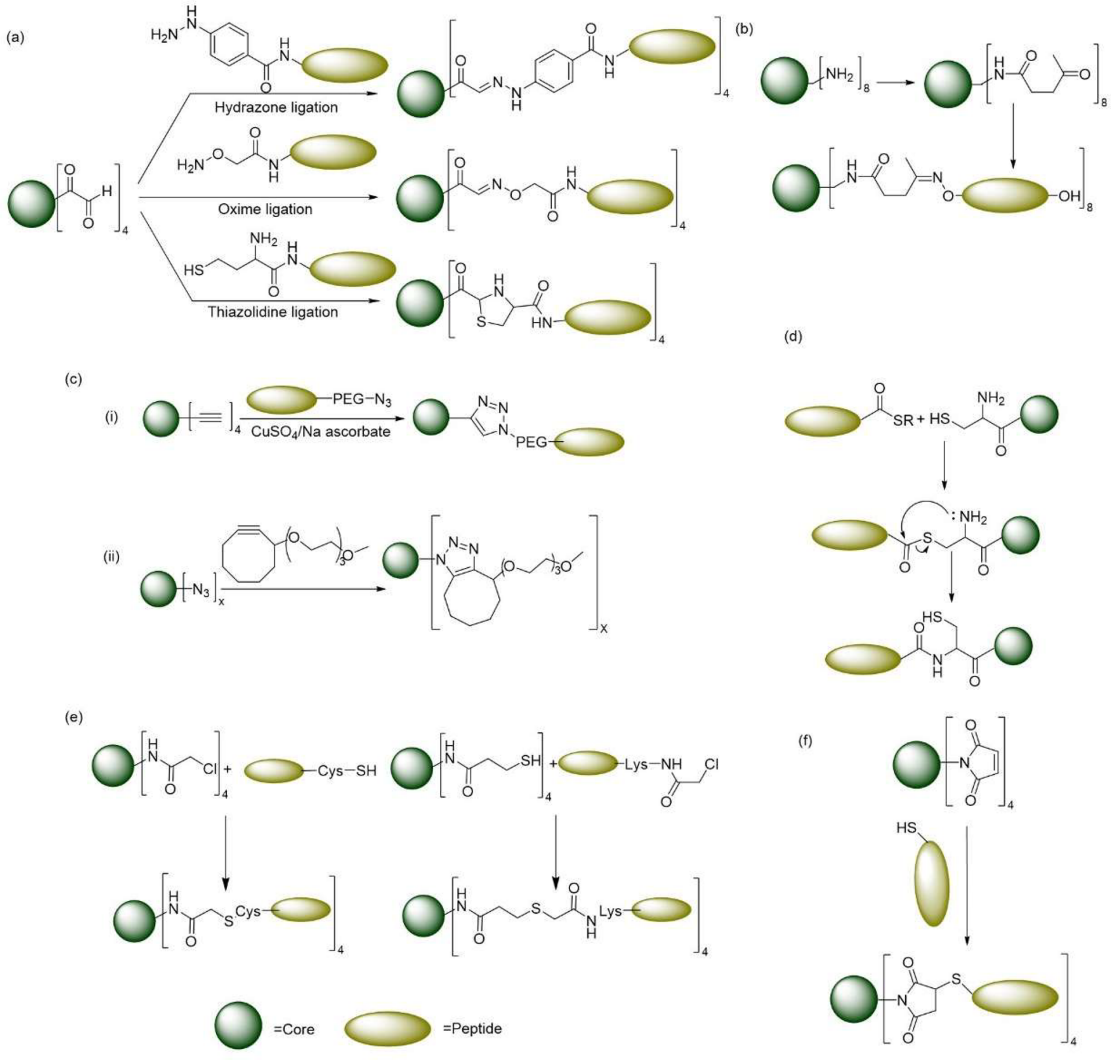
Cartoon illustrations of (a) the steps of hydrazone, oxime, and thiazolidine ligations; (b) a two-step ligation process developed by Mitchel; (c) azide–alkyne cycloaddition reactions employed to form dendrimers: (i) CuAAc-based and (ii) catalyst-free SPAAc-based procedures; (d) native chemical ligation (NCL) between cysteine at the N-terminal and a thioester group; (e) NCL between a cysteine terminated PPI dendrimer and an MPAL–thioester peptide; and (f) cysteine reacted with a maleimide functionalized dendrimer.

The concept of attaching peptides onto a dendrimer surface is known as multiple antigenic peptide (MAP) and was first discovered in 1988.^212^ Through various convergent modes of synthesis, unprotected peptides are attached to dendrimer surfaces. Peptides with 9–16 amino acids were attached to a PLL dendrimer core with the help of hydrazine, oxime, and thiozolidine ligation processes (Figure 3a). For hydrazine ligation process, a monohydrazide succinyl group was used; for oxime ligation, an (aminooxy)acetyl group was used; and for thiazolidine ligation, a cysteine was attached to the N-terminal of the peptide chain, and all the reactions were carried out in acidic conditions of pH 4–5. Although all processes were successful, thiazolidine ligation seemed to give better yields than the other methods.^225^ This thiazolidine ligation process was further modified^226^ to effectively synthesize peptide dendrimer-based vaccines against *Plasmodium falciparum*. This process proceeded faster and with a greater yield than those of the others. This ligation process was employed to synthesize peptide dendrimers with cores ranging from PAMAM to PLL conjugated to peptides containing cysteine at the N-terminal.^227,228^ A different two-step ligation process was later developed^229^ to conjugate peptides with ketone modified PAMAM dendrimers (Figure 3b).

## Click Chemistry for Dendrimer Synthesis

Another effective way of synthesizing MAP dendrimers was developed in 2005^230^ by employing the use of a microwave-assisted cycloaddition reaction. This reaction is based on a Huisgen 1,3-dipolar cycloaddition reaction between an azide and an alkyne to form [1,2,3]-triazole.^231^ Here the dendrimer surface were modified with alkyne groups, followed by coupling azido-terminated peptides onto the dendrimer surface with the help of microwave-assisted cycloaddition reaction. This approach is also known as “click chemistry”.^232^ This made the synthesis of di-, tri-, tetra-, and hexadecavalent peptide dendrimers possible, where the peptides themselves could range from small to large to cyclic.^233−236^ A copper catalyzed variation of this cycloaddition was later developed.^237^ This copper(I) catalyzed azide–alkyne cycloaddition (CuAAc) (Figures 3c(i)) has since then been widely used in history as a form of “click chemistry” for various bioconjugation reactions, including peptide conjugation to dendrimers.^238−240^ Although the Cu(I)-based catalyst has versatile applications, its cytotoxicity is a concern. Hence, an alternative approach known as the strain-promoted azide–alkyne cycloaddition (SPAAc) reaction was developed in catalyst-free conditions.^241^ A alkyne group is present on a cyclooctane to react with the azide group and readily release the bond strain, which is the major driving force behind the SPAAc reaction (Figure 3c(ii)).^242^ SPAAc was utilized for ligating dendrimers with azido-functionalized peptides as well as the ligation of azido-PEG (polyethylene glycol) dendrimer scaffolds with cyclooctyne functionalized peptides. The latter leads to the synthesis of 3D polymeric hydrogel scaffolds.^241−243^

## Cysteine-Based Native Chemical Ligation

The process of native peptide backbone formation, which enabled the ligation of two unprotected peptide chains together, was first discovered in 1994.^244^ Namely, a thioester at the α-carboxylic terminal of a peptide reacts with cysteine at the N-terminal of another peptide. This reaction results in the formation of a thioester bond, which subsequently undergoes rearrangement leading to the formation of a peptide bond between two unprotected peptide chains (Figure 3d).^244^ This native chemical ligation (NCL) chemistry is often utilized to synthesize convergently large proteins such as human lysozyme, chemokine IL-8, and human HIV-1 with complete biological activity.^245−247^ This strategy is also used to functionalize cysteine-terminalized poly(propyleneimine) (PPI) dendrimers with mercapto-propionic acid leucine (MPAL)-thioester peptides. It can also be used to generate MAP with various functionalities such as biotin or chromophores, which can help in bioimaging.^248,249^

Another process of cysteine-based ligation involves reacting the chloro-acetylated PAMAM dendrimer core with a cysteine terminated peptide at the C-terminal to form a thioether under mild conditions (Figure 3e). As a result, tetra- and octamer peptide dendrimers are formed. However, synthesizing higher generations becomes difficult.^228,250^ Another example of using the C-terminal cysteine for the coupling reaction is by reacting it with a maleimide terminated tetravalent PAMAM dendrimer (Figure 3f). Addition of very long peptides to dendrimers at a very mild condition was hence possible.^251−254^

Unfortunately, conjugating cyclic peptides to dendrimers can be challenging due to the significant steric strain involved. Both types of cyclic peptides, i.e., the head-to-tail cycles wound the ones containing disulfide bonds, have been studied for their potential to be conjugated with dendrimers to enhance their respective activities.^255,256^ From all these synthetic studies, it can be noted that conjugations of peptides, both linear and cyclic, are possible via various convergent methods. However, the synthesis of a higher generation of peptide dendrimers is still quite challenging, and further studies need to be carried out to improve the conjugation of a higher number of peptides onto the dendrimer surface.

## Machine Learning Approaches toward Designing Dendrimers

Just like with AMPs, machine learning models have been applied to the design of dendrimers as well.^257^ Developing dendrimers with possible biomedical applications includes predicting the encapsulation efficiency of small molecules,^258^ predicting its cytotoxicity,^259^ or the possibility of binding of a dendrimer to a biomolecule.^260^ Researchers use previous knowledge of dendrimer structure–activity relationships to fine-tune the designing of new dendrimers for their desired functions.^261^ For example, a fourth-generation glutamic acid dendrimer can encapsulate a single fluorophore for imaging purposes without producing cytotoxicity.^262^ Such technological advances and approaches will help develop noncytotoxic dendrimers with desired activities, eliminating the need for time-consuming chemical approaches.

## Peptide Dendrimers as Antibacterial Agents

Although linear peptides are biologically less toxic and superior to organic molecules in terms of reaching targets and penetrating cells, they have the inherent problem of being quickly digested through proteolysis. This problem often leads to a low bioavailability and low antibacterial efficacy. Also, the ability of some bacteria to form biofilms results in the generation of antimicrobial resistance.^263^ With the formation of biofilms, bacteria have 100–1000 times more tolerance toward antibiotics. Therefore, linear and cyclic peptides may become ineffective.^264^ These problems of proteolysis and bioavailability can be overcome with the use of peptide dendrimers.^4,11,265^ Although the branched structure of the peptide dendrimer looks similar to that of protein, serum proteases fail to recognize them as peptide or protein; hence, they can escape lysis by proteases. Therefore, *in vivo* stability and hence bioavailability are improved. The large dendritic structure also helps with spanning the bacterial membrane for easy pore formation because of a higher local concentration of bioactive structures, which is generally difficult for a single short peptide chain.^266^ Thus, peptide dendrimers have prolonged antibacterial activity and the capability to prevent biofilm formation. Consequently, bacterial strains that peptide dendrimers combat find it challenging to develop drug resistance.^267−269^

## Antibacterial Peptide Dendrimers

**Figure 4:**
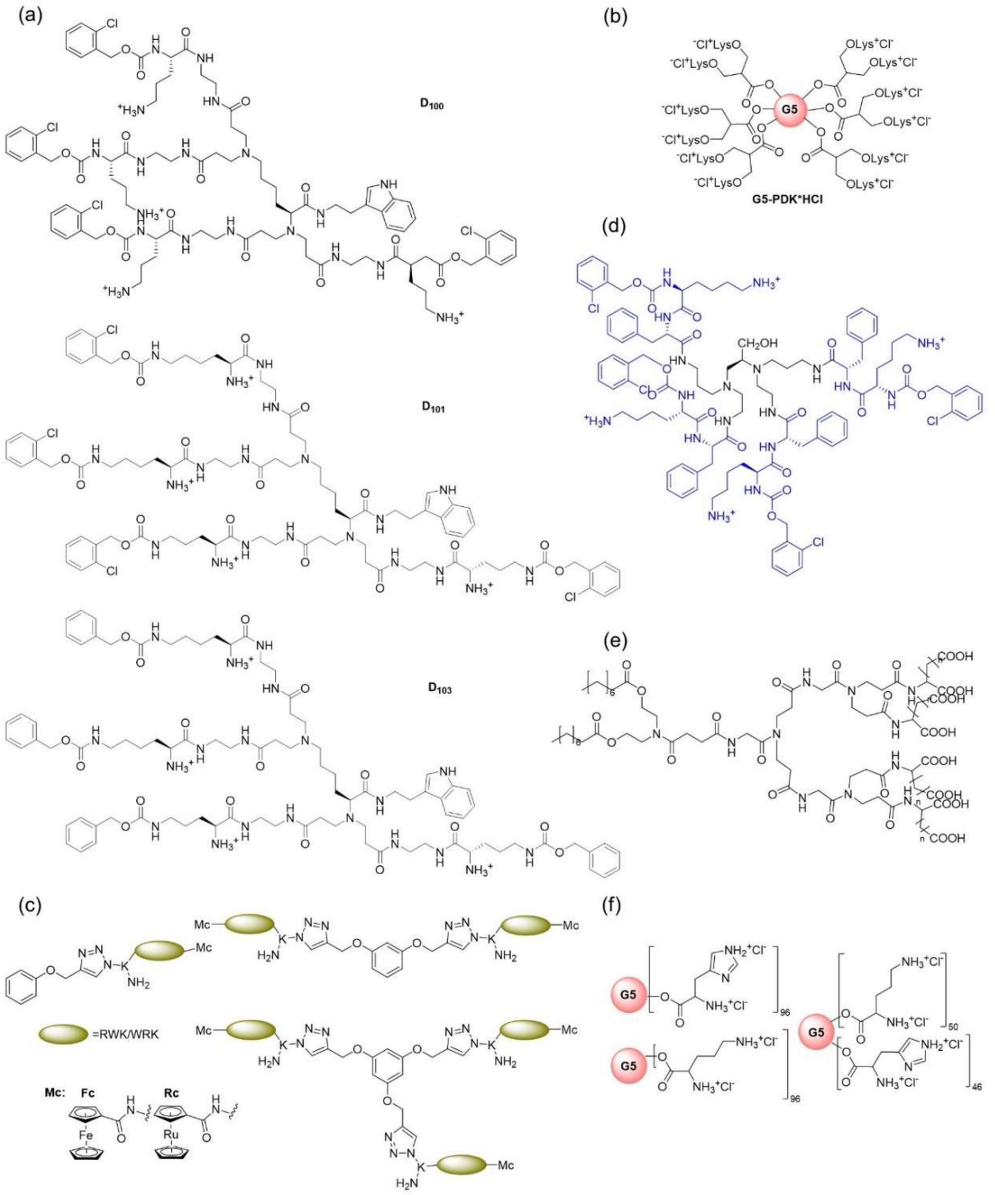
Structures of antibacterial peptide dendrimers: (a) D100, D101, and D103, (b) G5-PDK*HCl with a polyester dendrimer core, (c) peptide dendrimer containing metallocene synthesized via CuAAc click chemistry, (d) polyfunctional peptide dendrimer with well-spaced-out hydrophobic groups, (e) G1 dendrimer with aspartic, glutamic, or myristic acid terminals, and (f) G5H, G5K, and G5HK.

Peptide dendrimers possess a traditional dendrimer as the core and conjugate polypeptide chains with variable lengths on their surface. For example, D100, D101, and D103 (Figure 4a) are peptide dendrimers where the core is prepared by a method developed by Tomalia where lysine is used in place of amine,^270^ or G5-PDK (Figure 4b), which is a lysine terminated peptide dendrimer with an uncharged polyester-based dendrimer scaffold.^267,271^ There are also metallocene (Fe/Ru) containing peptide (WRK or RWK) dendrimers (Figure 4c) that are mono-, di-, or trivalent conjugations to an acetylene-based core conjugated via microwave-assisted CuAAC click chemistry, which falls under this category.^272^Table 2 lists some examples in detail about these types of peptide dendrimers.

**Table 2:** Examples of Antibacterial Peptide Dendrimers

dendrimer	structure	target bacteria	mechanism of action	ref
D100, D101, D103 (Figure 4a)	core is prepared by a method developed by Tomalia using lysine in place of amine	*S. aureus*	bacteriostatic effect due to membrane disruption	(270)
G5-PDK (Figure 4b)	lysine terminated peptide dendrimer with uncharged polyester-based dendrimer scaffold	*Acinetobacter baumannii*	positive charges on dendrimer surface due to protonation (from mild acids during Krebs cycle) helps bind to phosphate groups of genetic materials and membrane phospholipids, causing membrane disruption and cell lysis	(267, 271)
metallocene-based dendrimer (Figure 4c)	mono-, di-, or trivalent metallocene (Fe/Ru) containing peptide (WRK or RWK) dendrimers with a acetylene-based core conjugated via microwave-assisted CuAAC click chemistry	Gram-positive bacteria	rapid accumulation/absorption of the dendrimer into the bacterial cell	(272)
Figure 4d	dipeptide Phe-Lys with a 2-chlorocarbobenzoxy protection attached to a tetrafunctional novel dendrimer core	*S. aureus*, *E. coli*, and *Bordetella bronchiseptica*	well-spaced-out hydrophobic and hydrophilic groups interact with the negative cell wall, causing rupture	(273)
Figure 4e	G1 dendrimers with terminals of aspartic/glutamic acid and myristic acid	Gram-positive and Gram-negative bacteria	amphipathic nature of the anionic dendrimers	(274)
SSP-PAMAM-NH_2_	PAMAM dendrimer decorated with salivary Statherin inspired peptide sequence DDDEEKC, used as a coating on hydroxy apatite (HA)	*S. aureus* and *E. coli*	cell membrane and peptidoglycan layer disruption by PAMAM after DDDEEKC anchors positively charged PAMAM to HA surface	(268)
G5H, G5K, G5HK	PAMAM dendrimers decorated with histidine or lysine or both histidine and lysine	nonfermenting nosocomial Gram-negative bacteria	cell death by pore formation and creation of permeability issues by displacing Ca^2+^ and Mg^2+^ ions	(267)
calix[4]resorcinaranes containing peptide dendrimer	calix[4]resorcinarane derived from resorcinol is the core with four peptide molecules attached at four corners. peptides used were RLLR: AMP buforin 32–35 and RRWQWR: AMP bovine lactoferricin LfcinB 20–25	MDR strain of *E. coli* and vancomycin sensitive *S. aureus* strains	membrane disrupting ability of lysine, arginine, and tryptophan	(275)

The peptides used for the preparation of antibacterial dendrimers frequently possess tryptophan, arginine, and lysine to provide cationic properties as well as leucine to maintain amphiphilicity for reasons stated above.

## Antibacterial MAP Dendrimers

**Figure 5:**
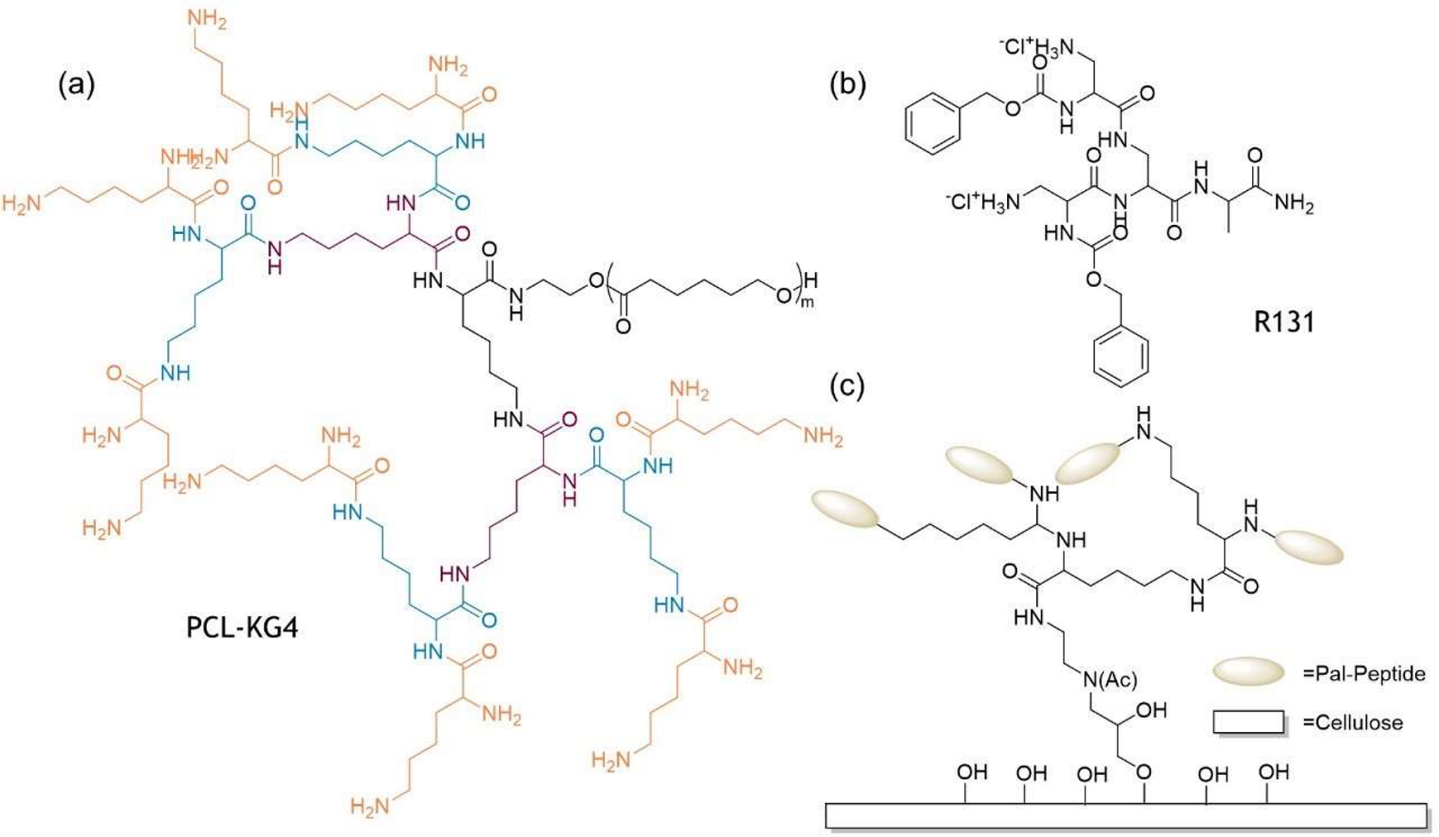
Antibacterial peptide dendrimers with PLL core: (a) PCL-K_G4_, each color representing different generations; (b) R131; and (c) peptides attached to a PLL dendrimer which is further attached to cotton fabrics.

The second form of antibacterial peptide dendrimers consists of amino acid branching units and surface peptidyl groups.^276,277^ Examples of such dendrimers are PCL-KG_4_ (Figure 5a),^278^ which is a PCL_20_ (polycaprolactone) containing PLL dendrimer, R131, [HClLys(2-Cl-Z)]_2_*Lys*-Ala-NH_2_ (Figure 5b), which is a divalent peptide dendrimer,^279,280^ (RLYR)_8_-([*K*_2_K]_2_*K*) and (RLYRKVYG)_8_-([*K*_2_K]_2_*K*), which are octameric peptide dendrimers with a PLL core,^281^ etc. Details of such peptide dendrimers with poly-lysine cores are given in Table 3.

**Table 3:** Peptide MAP Dendrimers with Poly-Lysine Cores

dendrimer	structure	target bacteria	mechanism of action	ref
PCL-KG_4_ (Figure 5a)	APCL_20_ (polycaprolactone) containing PLL dendrimer	broad-spectrum antibacterial effect against bacteria. excellent drug loading capacity for easy delivery	interaction and penetration of bacterial cell surface	(278)
R131 (Figure 5b)	divalent peptide dendrimer [HClLys(2-Cl-Z)]_2_*Lys*-Ala-NH_2_	both Gram-positive and Gram-negative bacteria	carpet mechanism due to electrostatic mode of interaction	(279,280)
(RLYR)_8_([*K*_2_K]_2_*K*) and (RLYRKVYG)_8_-([*K*_2_K]_2_*K*)	octameric peptide dendrimers with a PLL core	Gram-positive and Gram-negative bacteria	membrane disrupting ability, good proteolytic stability, and nontoxicity toward mammalian cells. therapeutic index >2200, 10-fold increase from linear peptide	(281)
Pal-X-Ala-d-Ala-X on PLL core, X = Lys, His, Arg (Figure 5c)	cationic peptide attached to PLL core, which in turn is covalently attached to cotton fabric	antibacterial fabric for wound dressing	membrane disruption due to electrostatic interaction	(282)
PEG-poly(Lys)	bottle-brush-like appearance of PEG-poly(Lys) coating on poly(styrene-*b*-(ethylene-*co*-butylene)-*b*-styrene) surface	96.83% effective on *S. aureus* and 99.99% on *E. coli*	membrane disruption and cell rupture due to electrostatic interaction and H-bonding of the brushes and bacterial membrane	(283)
(WKKIRVRLSA)_2_-*K*-8Aoc-NH_2_	dimeric peptide dendron synthesized by the SPPS method	Gram-positive bacteria, activity comparable to colistin and polymyxin B	membrane activity and lipid induced aggregation due to alternative cationic and hydrophobic structure	(284)
2D-24	tripeptide sequence RTtbR, modified from RWR, where 2,5,7-tri-*t*-butyl-L-tryptophan replaces tryptophan	persister cells and biofilms of *P. aeruginosa*	increased serum stability along with membrane depolarization and disruptive property	(285−287)

The most common amino acids to be used are lysine, arginine, and tryptophan, which impart better antimicrobial properties to these dendrimers. Also, the most common mode of action of these antibacterial dendrimers are membrane lysis and increased membrane permeability.^288,289^

## Antibacterial SPPS Dendrimers or Dendrons

**Figure 6:**
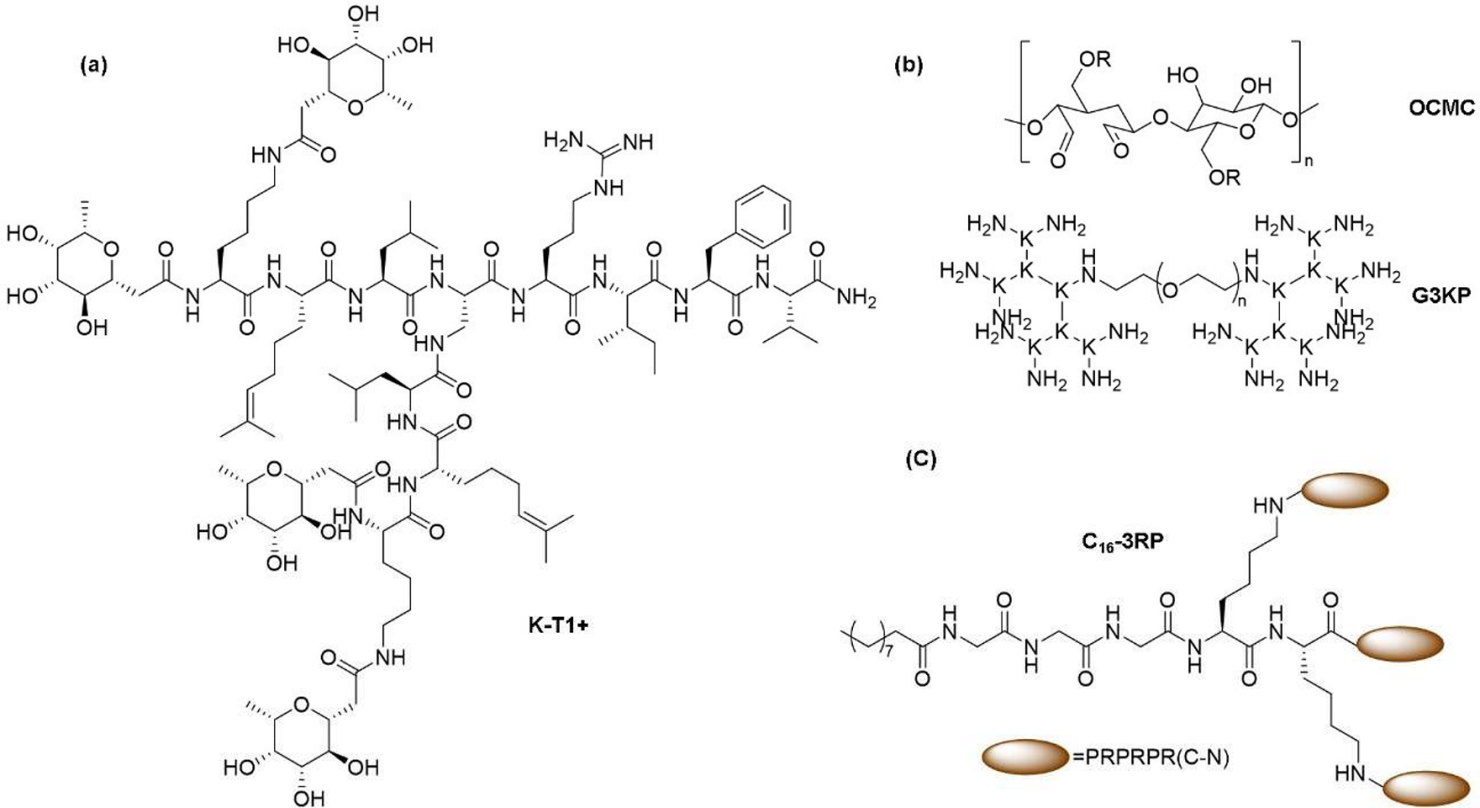
Structures of antibacterial peptide dendrimers. (a) K-T1+: (KRL)2 DapRIFV, (b) OCMC/G3KP, and (c) C_16_-3RP.

Last, but not least, comes the category of SPPS dendrimers (or dendrons) consisting of only amino acids. Examples of such types are (GalA-KPL)_4_(*K*FKI)_2_*K*HI-NH_2_,^290−292^ peptide dendrimer FD2, (C-Fuc-*Lys*ProLeu)_4_(*Lys*PheLysIle)_2_*Lys*HisIleNH_2_,^291,293,294^ or (CFuc-Lys- Pro)_8_(*Lys*LeuPhe)_4_(*Lys*LysIle)_2_*Lys*HisIleNH_2_,^295^ all of which have been synthesized by the SPPS method and have very good antibacterial properties. Details of such peptide dendrimers are given in Table 4 and Figure 6.

**Table 4:** Antibacterial Peptide Dendrimers or Dendrons Prepared by the SPPS Method

dendrimer	structure	target bacteria	mechanism of action	ref
(GalA-KPL)_4_(*K*FKI)_2_*K*HI-NH_2_	a multivalent galactosylated lectin LecA inhibitor peptide dendrimer containing 4-carboxypropyl-β-galactoside (GalA)	*P. aeruginosa*	preventing biofilm formation in *P. aeruginosa* by deactivating LacA	(290−292)
FD2	(C-Fuc-*Lys*ProLeu)_4_(*Lys*PheLysIle)_2_*Lys*HisIleNH_2_	*P. aeruginosa*	prevents biofilm formation in k by binding and deactivating lectin LecB	(291,293, 294)
D-FD2	the same peptide dendrimer containing d-amino acids			
(CFuc-Lys-Pro)_8_(*Lys*LeuPhe)_4_(*Lys*LysIle)_2_*Lys*HisIleNH_2_	C-fucosyl group containing glycopeptide dendrimer	*P. aeruginosa*	acts as fucose and prevents biofilm formation by binding to fucose specific lectin, LecB	(295)
(KRL)_2_DapRIFV (Figure 6a)	C-fucosylated dimeric peptide dendrimer	*P. aeruginosa*	prevents biofilm formation by binding to fucose specific lectin PA-IIL	(296)
(*b*-Gal-OC_6_-H_4_CO-Lys-Pro-Tyr)_4_(*Lys*-Phe-Lys-Ile)_2_-*Lys*-His-Ile-NH_2_	tripeptide modification of the previously found lectin LecA inhibitor GalAG2, containing the tripeptide sequence KPY	*P. aeruginosa*	enhanced binding ability to LecA, hence preventing biofilm formation	(297)
G3KL	(KL)_8_(*K*KL)_4_(*K*KL)_2_*K*KL is a lysine- and leucine-based peptide dendrimer	*P. aeruginosa*, *A. baumannii*, and *E. coli*	membrane disruption due to electrostatic binding to bacteria cell membrane	(290−302)
TNS18	(OF)_4_(*K*BL)_2_*K*KLK(C10) is a C-terminal lapidated peptide dendrimer	MDR Gram-negative bacteria, i.e., *P. aeruginosa* and *A. baumannii*, and MRSA	hydrophobically collapsed structure unfolds in contact with bacterial membrane followed by membrane disruption	(303−305)
OCMC/G3KP (Figure 6b)	bioadhesive based on a polysaccharide OCMC and peptide dendrimer G3KP	*E. coli* and *S. aureus*	electrostatic interaction between bacterial membrane and protonated amines in polylysine-based G3KP with a PEG tail	(306)
		useful for wound healing and effective both *in vitro* and *in vivo*		
C_16_-3RP (Figure 6c)	self-assembling peptide dendrimer nanoparticles (SPDNs)	broad spectrum antibacterial effect	inhibition of ribosomal biogenesis for influencing energy generation. no apparent resistance development for this SPDN	(307)
(KLK)_2_*K*LLKLL-NH_2_	lysine- and leucine-based peptide dendrimer, synthesized by the SPPS method	Gram-negative bacteria	membrane lysis due to high positive charge density. TI: 70–235	(308)
(RWRW)_4_*K*KβA-NH_2_	indolicidine influenced peptide dendrimer (RWRW)_4_*K*K***β***A-NH_2_	MDR strains of bacteria with activity comparable to conventional antibiotics	membrane lysis due to alternate cationic and hydrophobic groups. little resistance generation even after 400 cycles	(269)
(Ahx-L)_8_(*K*KL)_4_(*K*KL)_2_*K*KL, (Ahx-L)_8_(*K*KL)_4_(*K*KLL)_2_*K*KKL, (Ac-KL)_8_(*K*K L)_4_(*K*KL)_2_*K*KL, and (Ac-KL)_8_(*K*KL)_4_(*K*KLL)_2_*K*KKL (Ahx= aminohexanoic acid)	lysine- and leucine-based peptide dendrimer with high positive charge	drug-resistant bacteria	pH dependent membrane lytic activity	(309)

The repeated use of leucine and lysine is observed for most AMPDs to maintain amphiphilicity and the cationic nature of the dendrimer to achieve the best possible efficacy for these cationic dendrimers, the most common mode of action being membrane disruption.

## Challenges to the Use of Peptide-Based Dendrimers in Antibacterial Applications

However, researchers still need to optimize many aspects in studying the use of dendrimers as antimicrobial agents. First, even though the interaction efficiency increases in dendrimers with increasing multivalency, it has been noticed that the number of dendrimers that can interact with the bacterial membrane decreases with an increase in the generation of dendrimers. The toxicity of antibacterial dendrimers also increases as the dendrimer size and generation increase.^187,310^ Moreover, the electrostatic forces, which operate between the negatively charged bacterial membrane and the positively charged dendrimers, peptide dendrimers, or dendrons, make them more effective against Gram-negative bacteria than Gram-positive bacteria.^311^

The positive charge nature of these dendrimers also makes them toxic toward mammalian cells. Also, dendrimers in a higher generation tend to be create more immunogenic responses.^267,312−315^ From the synthesis point of view, complete purification of peptide dendrimers is still a challenge as the removal of single modification or single removal product is difficult with conventional methods like gel chromatography or reverse-phase high performance liquid chromatogrpahy.^187^ A 1% defective compound or impurity in the first generation is likely to lead to almost 25% of defective impurities reaching the fifth-generation synthesis, at which point purification becomes rather difficult.^315^ Also, while attaching peptides on organic dendrimers, it is often noticed that 100% modification of the surface functional groups is not possible; only a few peptides can be attached.^316,317^

**Figure 7:**
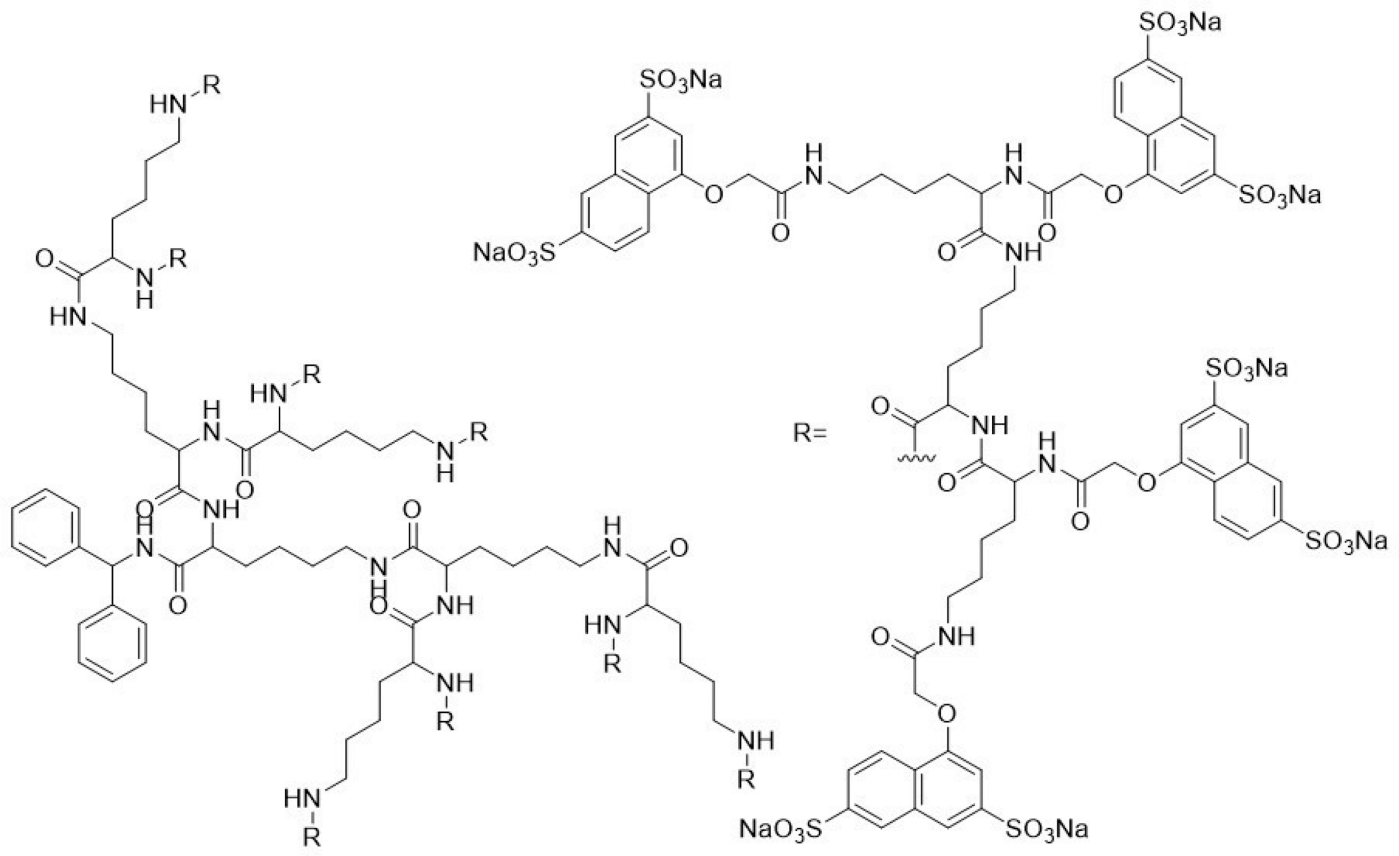
Chemical structure of VivaGel, the AMPD currently in clinical use.

Nevertheless, peptide dendrimers have proved to be interesting molecules, which can effectively combat the problem of growing resistance generation in pathogenic bacterial species. Lowering the MIC values is made possible by the multivalency of peptide dendrimers, which enables considerably more concentrated drug contact with the bacterial surface. With a high TI value, peptide dendrimers should be safe to use in clinical settings.^281,308^ Peptide dendrimers are becoming widely recognized as “gold-standard reagents” in the field of developing antibacterial drugs due to these distinctive characteristics. They are an emerging class of antibiotics already being used in clinical settings. One such example is VivaGel, a poly-l-lysine dendrimer already approved in Australia and Europe for the treatment of bacterial vaginosis (Figure 7).^318,319^ However, new methods or strategies need to be explored to address certain issues such as reducing mammalian cell toxicity and improving the purification of dendrimer products. Also, the problem of discrepancy between *in vivo* and *in vitro* efficacy, which prevents most drugs from entering the market, needs to be overcome in order to tackle the rising resistance development in bacteria.

## Conclusions

Due to the unique mode of action of bacteria and their capability of quick resistance generation, new antimicrobials continue to fail in showing prolonged effects. Although peptide-based antibacterial agents appear to be a safer alternative due to their biocompatibility and varied modes of action, they are quickly destroyed *in vivo* due to the presence of proteases that break down peptide bonds. It is observed that the multivalency generated upon conjugating peptides onto nanoparticles increases their efficacy by several folds. These nanoparticle conjugated peptides are easier to synthesize as well as purify compared to conventional methods of synthesis. However, the toxicity of the metal nanoparticles became a hindrance for their effective *in vivo* applications. Hence, dendrimers came into rescue. Dendrimers have better bioactivity and more biostability but less biocompatibility due to their multivalent macromolecular structure. Hence, combining the features of biocompatibility of peptides and biostability of dendrimers to form peptide dendrimers and glycopeptide dendrimers has created an improved new candidate for antibacterial compounds. With the emergence of newer methods to synthesize peptide dendrimers, it is now possible to use their versatility and multifaceted mode of action to generate better antibiotics while preventing bacteria from gaining fast resistance to these macromolecular antibacterial compounds. The multivalency of peptide dendrimers, like peptide–nanoparticle conjugates, allows much more concentrated interaction of drugs with the bacterial surface, which, in turn, helps in lowering the MIC values. This in turn increases the therapeutic index value for these drugs, lowering the cytotoxicity of the compounds. Also, the absence of metals makes them more suitable for *in vivo*-based applications. Because of these unique features, peptide dendrimers are gaining widespread acknowledgment as “gold-standard reagents” in the field of antibacterial drug development. Figure S2 shows the summarized bar graphs of the references cited in this review paper. An increasing number of studies related to antibacterial peptides and peptide dendrimers have been published in the past decade. Nevertheless, significant work still needs to be done in this field to ensure that peptide dendrimers can be successfully marketed as a new class of antibacterial drugs. Newer fields of research are emerging continuously for developing better organic mimics of metal nanoparticles to preserve the multivalency of peptide conjugates on top of ensuring ease of synthesis. Such examples are the use of graphene oxide, or fullerenes, or other multivalent organic cores, with exposed functional groups for peptide ligation. Machine learning models are employed, as well, to generate optimized peptide dendrimers with better efficacy. These peptide-based supramolecular structures are slowly changing the perspective of antibacterial drugs as effective and safe alternatives to small-organic-molecules-based antibiotics. More and more research should be carried out to reach the ultimate goal of preventing antibiotic resistance. The current review article is focused on antibacterial peptide dendrimers. However, we have observed that similar designs and killing mechanisms with antifungal peptide dendrimers against *Candida* spp. have been investigated (Table S1). Thus, we believe that the structures and amino acid sequences used in the design of antibacterial peptide dendrimers could also be suitable for inhibiting the growth of organisms other than bacteria.

## Supporting Information Available

List of antifungal peptide dendrimers; chemical structures of some antifungal peptide dendrimers; and distribution of the published years of the references cited in this paper (PDF)
