# Correction to “Peptide Dendrimer-Based Antibacterial Agents: Synthesis and Applications”

**DOI:** 10.1021/acsinfecdis.6c00594

**Published:** 2026-07-14

**Authors:** Suchita Paul, Sandeep Verma, Yu-Chie Chen

**Affiliations:** † International College of Semiconductor Technology, 34914 National Yang Ming Chiao Tung University, Hsinchu 300, Taiwan; ‡ Department of Chemistry, Indian Institute of Technology Kanpur, Kanpur 208016, Uttar Pradesh, India; § Gangwal School of Medical Sciences and Technology, Indian Institute of Technology Kanpur, Kanpur 208016, Uttar Pradesh, India; ∥ Department of Applied Chemistry, National Yang Ming Chiao Tung University, Hsinchu 300, Taiwan

The first affiliation for Suchita Paul and Yu-Chie Chen should be International College of Semiconductor Technology and not Institute of Semiconductor Technology. The corrected affiliation is reflected in the Author Information of this Correction.

