# Supplementary material for: Peptide Dendrimer-Based Antibacterial Agents: Synthesis and Applications
Source: ACS Infect Dis. 2024 Mar 1;10(4):1034–55. doi: 10.1021/acsinfecdis.3c00624 (PMC11019562; doi:10.1021/acsinfecdis.3c00624)
Supplement: Supplementary file 1 — id3c00624_si_001.pdf [file id3c00624_si_001.pdf]

Supporting Information

Peptide Dendrimer-based Antibacterial Agents: Synthesis and Applications

Suchita Paul,[a,b] Sandeep Verma,[b,c*] and Yu-Chie Chen[a,d*]

[a] Institute of Semiconductor Technology, National Yang Ming Chiao Tung University, Hsinchu 300, Taiwan

[b] Department of Chemistry, Indian Institute of Technology Kanpur, Kanpur 208016, Uttar Pradesh, India

[c] Gangwal School of Medical Sciences and Technology, Indian Institute of Technology Kanpur, Kanpur 208016, Uttar Pradesh, India

[d] Department of Applied Chemistry, National Yang Ming Chiao Tung University, Hsinchu 300, Taiwan

*Corresponding authors

Sandeep Verma

E-mail: sverma@iitk.ac.in

Tel: +91-512-6797643

Yu-Chie Chen

E-mail: yuchie@nycu.edu.tw

Tel: +886-3-5131527

Table S1. List of antifungal peptide dendrimers

Peptide dendrimer	Structure	Target fungi	Mechanism of action	Ref.
$(RLYR)_8([K_2K]_2K)$ and $(RLYRKVYG)_8$-$([K_2K]_2K)$	These are octameric peptide dendrimers with a PLL core	*Candida albicans, C. kefyr, C. triopicalis*	Membrane disrupting ability.	1
**Fig. S1a**	PPL dendrimer core capped with other amino acids and blocked N terminals	*C. albicans*	Interaction between cationic dendrimers and negatively charged fungal cell membranes.	2
R131 **(Fig. 5b)**	A divalent peptide dendrimer [HClLys(2-Cl-Z)]$_2$*Lys*-Ala-NH_2	*C. albicans*	The carpet mechanism of cell membrane disruption due to electrostatic mode of interaction.	3, 4
R-isomer of R131	The R-isomer of the peptide dendrimer [HClLys(2-Cl-Z)]$_2$*Lys*-Ala-NH_2	*C. albicans*	The carpet mechanism due to the electrostatic mode of interaction between positive amino acids and negative bacterial membranes.	4
D100, D101, D103 **(Fig. 4a)**	Peptide dendrimer where the core is prepared by method developed by Tomalia where lysine is used in place of amine.	*C. albicans*, *C. krusei, C. parapsilosis*	Electrostatic interaction between positive dendrimer and negative fungal cell membrane leading to membrane swelling, disruption, and cell death.	5
PLL based dendrimer with long lipophilic chains.	Cationic lipopeptides with PLL dendron with amphiphilic polar heads and C8 or C12 chain at the C terminal.	*Candida* spp.	Inhibition of 1,3-β-D-glucan synthase and its incorporation in the cell wall synthesis.	6
D186	A modified PLL dendrimer with N,N-	*Candida* spp.	Impairs adhesive properties of fungi while tryptophan	7, 8

	dioctyl tail and four tryptophan residues.		based terminals help with further disruption of fungal cell membranes.	
**Fig. S1b**	A PAMAM based dendrimer with a lysine core, terminal Tryptophans and a C12 chain at the N terminal of the Lysine.	*Candida spp.*	Fungal cell apoptosis due to impaired cell wall formation and interfering with chitin synthesis.	8
**Fig. S1c**	A modified G1 dendrimer with a C12 lipophilic chain at the C-terminal.	*Candida* spp.	The amphiphilic nature of the peptide dendrimers is responsible for permeation into the fungal cells.	9
**Fig 4d**	Dipeptide Phe-Lys with a 2-chlorocarbobenzoxy protection attached to tetrafunctional novel dendrimer core.	*C. albicans*, *C. parapsilosis,* and *C. krusei*	Well-spaced out hydrophobic and hydrophilic groups interacts with the negative cell wall causing rupture.	10
Calix[4]resorcinaranes containing peptide dendrimer	Calix[4]resorcinarane derived from resorcinol is the core with four peptide molecules attached at four corners. Peptides used were RLLR: AMP buforin 32-35 and RRWQWR: AMP bovine lactoferricin LfcinB 20-25.	*C. albicans, C. glabrata, C. tropicalis, C. parapsilosis* and *C. krusei*	It is speculated that mechanism of action is membrane disruption due to electrostatic interaction between dendrimer and fungal cell membrane.	11

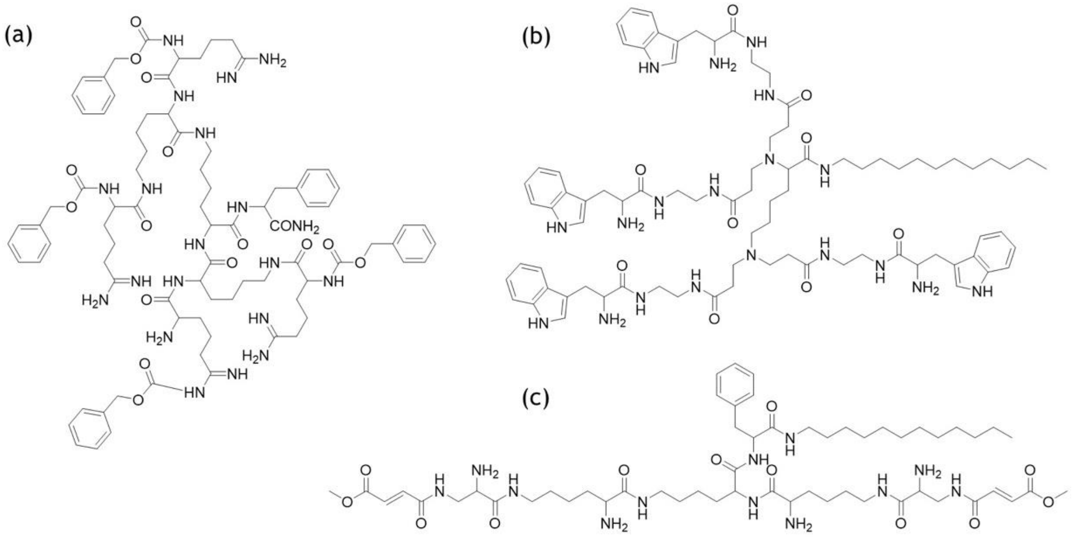

Figure S1. Chemical structures of some antifungal peptide dendrimers

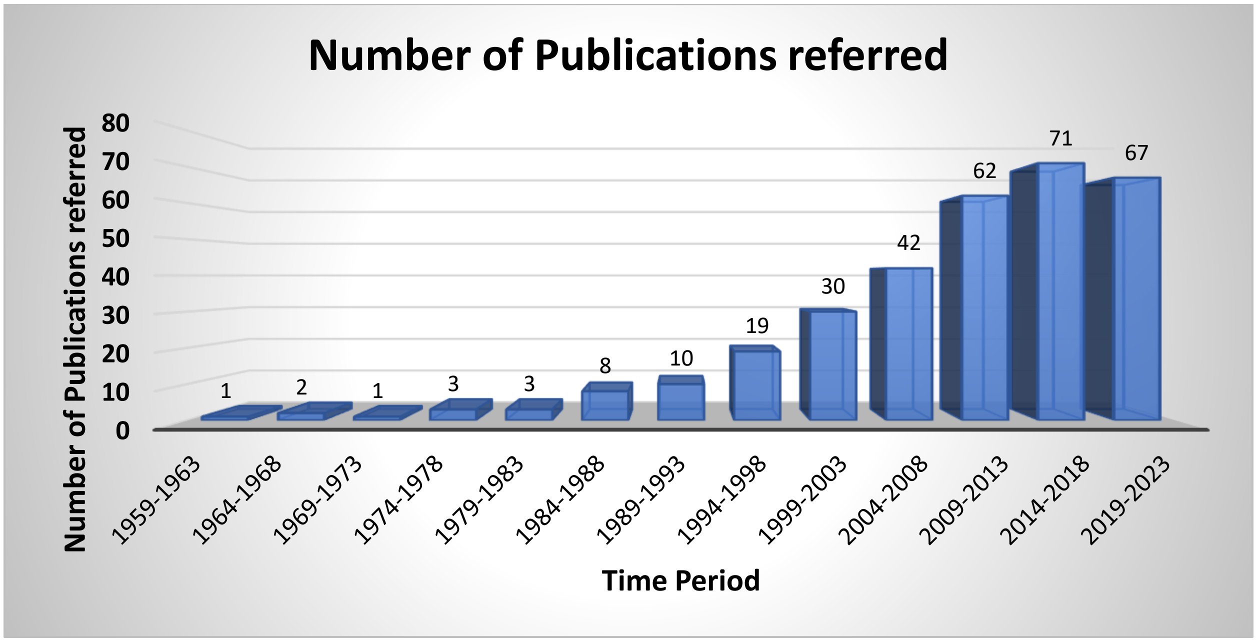

Figure S2. Distribution of the published years of the references cited in this paper.

References

(1) Tam, J. P.; Lu, Y. A.; Yang, J. L. Antimicrobial dendrimeric peptides. *Eur. J. Biochem.* **2002**, *269* (3), 923-932.

(2) Janiszewska, J.; Urbańczyk-Lipkowska, Z. Synthesis, antimicrobial activity and structural studies of low molecular mass lysine dendrimers. *Acta Biochim. Pol.* **2006**, *53* (1), 77-82.

(3) Klajnert, B.; Janiszewska, J.; Urbanczyk-Lipkowska, Z.; Bryszewska, M.; Shcharbin, D.; Labieniec, M. Biological properties of low molecular mass peptide dendrimers. *Int. J Pharmaceut.* **2006**, *309* (1-2), 208-217.

(4) Janiszewska, J.; Urbanczyk-Lipkowska, Z. Amphiphilic dendrimeric peptides as model non-sequential pharmacophores with antimicrobial properties. *Microbial Physiol.* **2007**, *13* (4), 220-225.

(5) Lind, T. K.; Polcyn, P.; Zielinska, P.; Cárdenas, M.; Urbanczyk-Lipkowska, Z. On the antimicrobial activity of various peptide-based dendrimers of similar architecture. *Molecules* **2015**, *20* (1), 738-753.

(6) Janiszewska, J.; Sowińska, M.; Rajnisz, A.; Solecka, J.; Łącka, I.; Milewski, S.; Urbańczyk-Lipkowska, Z. Novel dendrimeric lipopeptides with antifungal activity. *Bioorg. Med. Chem. Lett* **2012**, *22* (3), 1388-1393.

(7) Staniszewska, M.; Bondaryk, M.; Zielińska, P.; Urbańczyk-Lipkowska, Z. The in vitro effects of new D186 dendrimer on virulence factors of Candida albicans. *J. Antibiot.* **2014**, *67* (6), 425-432.

(8) Zielińska, P.; Staniszewska, M.; Bondaryk, M.; Koronkiewicz, M.; Urbańczyk-Lipkowska, Z. Design and studies of multiple mechanism of anti-Candida activity of a new potent Trp-rich peptide dendrimers. *Euro. J. Med. Chem.* **2015**, *105*, 106-119.

(9) Stolarska, M.; Gucwa, K.; Urbańczyk-Lipkowska, Z.; Andruszkiewicz, R. Peptide dendrimers as antifungal agents and carriers for potential antifungal agent—N3-(4-methoxyfumaroyl)-(S)-2, 3-diaminopropanoic acid—synthesis and antimicrobial activity. *J. Pep. Sci.* **2020**, *26* (1), e3226.

(10) Polcyn, P.; Jurczak, M.; Rajnisz, A.; Solecka, J.; Urbanczyk-Lipkowska, Z. Design of antimicrobially active small amphiphilic peptide dendrimers. *Molecules* **2009**, *14* (10), 3881-3905.

(11) Pineda-Castañeda, H. M.; Maldonado-Villamil, M.; Parra-Giraldo, C. M.; Leal-Castro, A. L.; Fierro-Medina, R.; Rivera-Monroy, Z. J.; García-Castañeda, Peptide-Resorcinarene Conjugates Obtained via Click Chemistry: Synthesis and Antimicrobial Activity. *J. Antibiot.* **2023**, *12* (4), 773.
